# Real‐world clinical impact of implementing and updating a deep learning‐based automatic contouring system in rectal cancer radiotherapy

**DOI:** 10.1002/acm2.70768

**Published:** 2026-08-28

**Authors:** Ningyu Wang, Tongzhen Xu, Yujie Kang, Yuting Liang, Wenyu Wang, Wensheng Nie, Jianrong Dai, Yuan Tang, Kuo Men

**Affiliations:** ^1^ National Cancer Center/National Clinical Research Center for Cancer/Cancer Hospital Chinese Academy of Medical Sciences Peking Union Medical College Beijing China

**Keywords:** automatic contouring, deep learning, rectal cancer radiotherapy, system implementation, system update

## Abstract

**Background:**

Accurate delineation of target volumes and organs‐at‐risk (OARs) is a critical yet labor‐intensive component of rectal cancer radiotherapy. While deep learning (DL)‐based automatic contouring systems are increasingly used to address inter‐observer variability and improve efficiency, artificial intelligence models require rigorous quality assurance and updates to reflect current technology. However, high‐level evidence regarding the longitudinal real‐world impact of implementing and iteratively updating these systems in clinical workflows is currently lacking.

**Purpose:**

This study aimed to evaluate the real‐world clinical impact of implementing and updating a DL‐based automatic contouring system in rectal cancer radiotherapy to generate high‐quality evidence of iterative updates.

**Methods:**

This longitudinal retrospective analysis included 150 patients divided into three cohorts: pre‐implementation (n1 = 50), post‐implementation (n2 = 50), and post‐update (n3 = 50). Geometric similarities between unedited‐automatic and final treatment contours were compared across cohorts. Failure rates were systematically analyzed. Six oncologists contoured 21 additional cases through manual, first‐generation (Auto1), and second‐generation (Auto2) system‐assisted methods to evaluate contouring time, inter‐observer consistency, and accuracy. Additionally, a 5‐point Likert scale was used by two blinded senior oncologists to assess the clinical acceptability of the generated contours.

**Results:**

The mean Dice similarity coefficient (DSC) values of clinical target volume (CTV) before and after implementing the automatic contouring system were 0.87 ± 0.04 and 0.88 ± 0.04 (*P* = 0.067), while those of OARs were 0.80 ± 0.06 and 0.88 ± 0.05 (*P* < 0.001), respectively. Following the system update, they improved from 0.88 ± 0.04 to 0.93 ± 0.04 for CTV (*P* < 0.001) and from 0.88 ± 0.05 to 0.95 ± 0.02 for OARs (*P* < 0.001). The system update achieved an approximately 80.6% reduction in the mean failure rate. Auto2‐assisted method decreased the total time by approximately 58.8% compared with the manual method, and 21.9% compared with the Auto1‐assisted method. This method also demonstrated optimal inter‐observer consistency (0.95 ± 0.03) and accuracy (0.94 ± 0.03) for CTV. In the blinded clinical evaluation, 99.2% (125/126) of the oncologist‐revised final contours received a Likert score of ≥ 4, and Auto2‐generated unedited contours showed significantly higher clinical acceptability than Auto1 (4.02 ± 0.25 vs. 3.26 ± 0.49, *P* < 0.001)

**Conclusions:**

Implementing an automatic contouring system provided crucial guidance for clinical practice. Its iterative update significantly reduced workload and inter‐observer variation while enhancing contouring efficiency and quality.

## 1 INTRODUCTION

Rectal cancer remains among the most common gastrointestinal malignancies worldwide, ranking high in both morbidity and mortality.^1^ Radiotherapy is critical in its multidisciplinary management, focusing on reducing local recurrence rates and downstaging tumors preoperatively.^2^ For treatment efficacy and normal tissue sparing, the target volume and organs‐at‐risk (OARs) must be delineated accurately.^3^

Traditionally, radiation oncologists manually contour these structures. According to rectal cancer radiotherapy guidelines,^4^, ^5^ gross tumor volume (GTV) includes the visible and palpable tumor in the rectum, as well as any metastatic lymph nodes, whereas the clinical target volume (CTV) includes the above‐mentioned GTV region plus high‐risk lymph node drainage areas and high‐risk recurrence areas, such as the mesenteric area and pelvic presacral region. Considering the substantial differences in tissue contrast between computed tomography (CT) and magnetic resonance imaging (MRI), dual‐modal imaging is needed to delineate target volumes for patients with rectal cancer. Generally, GTV delineation largely relies on MRI images to clearly visualize the primary tumor site,^6^ while CT images are primarily used for CTV and OARs delineation and plan designing. Overall, manual contouring is a labor‐intensive, time‐consuming process and is subject to inter‐observer and intra‐observer variability,^7^ likely compromising treatment quality and consistency.

Several automatic contouring techniques have been developed and integrated into clinical workflows to address such challenges. Earlier methods relied on atlas‐based auto‐segmentation (ABAS), which used a set of representative patients with carefully delineated structures and mapped these structures onto new patients through deformable registration. ABAS has already reduced workload in many radiotherapy departments, but given the anatomical differences, establishing a “universal atlas” was difficult.^8^, ^9^ Recently, deep learning (DL) methods have received widespread attention in medical imaging segmentation, offering improved performance over atlas‐based approaches.^10^, ^11^ Various studies horizontally compared geometric similarities, either quantitatively or qualitatively, between manual and automatic contours to validate the performance of automatic contouring algorithms.^12^, ^13^, ^14^, ^15^, ^16^ The influence of these two contour types on plan quality has also been assessed.^17^, ^18^, ^19^

According to recent joint guidelines on artificial intelligence (AI)‐assisted auto segmentation issued by ESTRO and AAPM,^20^ rigorous clinical validation and quality assurance are necessary for AI models. In real‐world clinical settings, simple geometric assessments are insufficient to validate the effectiveness of AI‐based auto‐segmentation. Moreover, most AI models are trained on data of a specific historical cohort. Subsequently, given the changes in patient data or the introduction of new work procedures in clinical practice, updating or upgrading the model may be necessary to reflect the current state‐of‐the‐art technology. Although previous research efforts have achieved promising advancements in auto‐segmentation based on the guidelines, high‐level clinical evidence remains lacking. Most investigations have focused on the horizontal comparison of the algorithm or commercial software against ground truth (GT), rather than longitudinal tracking in real‐world clinical practice after model implementation. Evidence regarding how algorithm iterative updates influence daily clinical workflows, oncologist workload, and the contour quality is also limited. Finally, the reasons of auto‐segmentation failure (e.g., contours exceeding control‐line deviations) have not been systematically examined.

To fill this evidence gap, we conducted a retrospective study involving 150 patients with rectal cancer who received preoperative radiotherapy in our institution from three phases: (i) pre‐implementation phase (when all treatment contours were manually generated); (ii) post‐implementation phase (following deployment of the first‐generation DL‐based automatic contouring system); (iii) post‐update phase (following deployment of the second‐generation system). We focused on a longitudinal comparison across these three phases. Additionally, oncologists’ editing burden, workflow efficiency, and inter‐observer consistency were quantified. Crucially, we systematically analyzed failure modes to determine why and when automatic contours exceeded control limits. This clinically oriented research directly responds to the guidelines’ call for ongoing validation of AI model updates and provides high‐level evidence on the real‐world impact of evolving auto‐segmentation technology.

## 2 MATERIALS AND METHODS

### 2.1 Automatic contouring system implementation and update

Before May 2018, our department had not implemented any automatic contouring system. Manual contours were delineated by radiation oncologists according to the international consensus guidelines on CTV delineation in rectal cancer.^4^

The first‐generation DL‐based automatic contouring system was implemented in May 2018, employing the DeepLab V3 architecture^21^ as its segmentation backbone. In December 2024, the system was updated to the second generation, replacing DeepLab V3 with the nnU‐Net V2 framework.^22^ Both models were trained as research prototypes on our institutional dataset using an in‐house server. In clinical practice, for each new patient, planning CT images are transferred from the treatment planning system (TPS) to the server, where the model performs automatic contouring. The predicted contours are then transmitted back to the TPS for review and manual adjustment by the radiation oncologist.

### 2.2 Study analysis and patient selection

**FIGURE 1:**
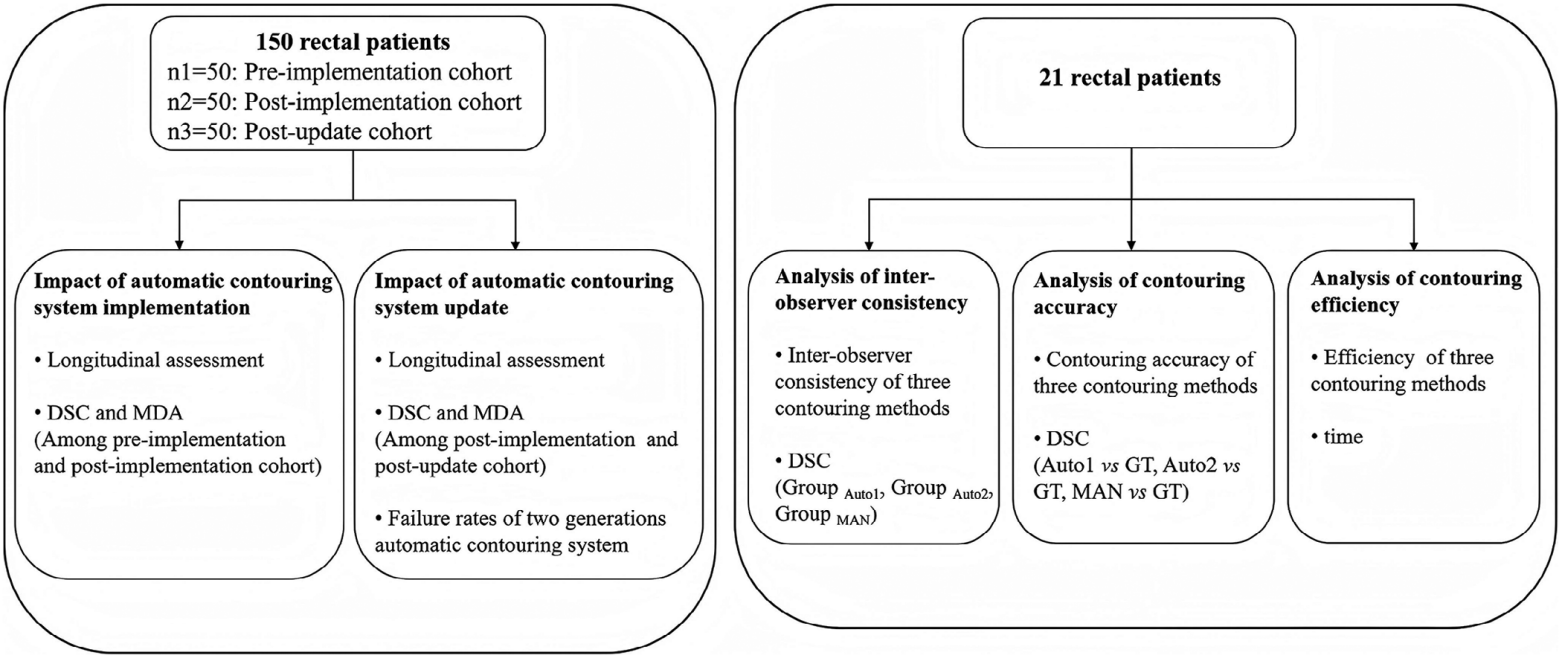
Overall schematic of the study. A total of 150 patients were used for comparisons among different cohorts and phases, while 21 were employed to assess inter‐observer consistency, accuracy, and efficiency of the three contouring methods. DSC, dice similarity coefficient; MDA, mean distance to agreement. DSC_Group _Auto1_, DSC_Group _Auto2_, and DSC_Group _MAN_ represent the inter‐observer similarities between two oncologists using the first‐generation automatic contouring system (Auto1)‐assisted method, the second‐generation system (Auto2)‐assisted method, and the manual contouring method, respectively. Likewise, DSC_Auto1, DSC_Auto2, and DSC_MAN quantify the similarities between the GT and the structures contoured by the oncologists using three methods.

This study was approved by the independent ethics committee of the National Cancer Center/Cancer Hospital, Chinese Academy of Medical Sciences. Figure 1 illustrates the specific research content of this study and is mainly divided into two parts.

The first component comprised a 150‐patient clinical‐impact dataset, which was divided into three cohorts corresponding to the three clinical phases. Its purpose was to assess the clinical effect of introducing and updating the automatic contouring system in routine practice. This part did not use GT contours as the reference standard. It evaluated whether the discrepancy between auto‐generated contours and the clinically accepted treatment contours decreased after implementation and updating, and it further examined auto‐segmentation failures. Baseline characteristics, including age, sex, clinical stage, and tumor distance from the anal verge, were compared among the three cohorts to assess potential confounding.

The second component was an independent 21‐patient performance‐evaluation dataset, in which all cases were re‐delineated by six oncologists using three contouring methods. This component was designed to provide a comparison of the three contouring approaches with respect to inter‐observer consistency, contouring accuracy, and efficiency. In this cohort, accuracy was evaluated against a predefined GT established by expert consensus.

The high‐risk lymph node drainage regions for the patients used in this study encompass mesentery area, pelvic presacral region, lateral cervical lymph node area and obturator lymph nodes area. Between 2018 and 2024, the CT imaging protocol for rectal cancer radiotherapy remained consistent at our institution: a slice thickness of 5 mm, a field of view (FOV) of 65 cm, a scanning range extending from at least the second lumbar vertebra to below the perineum, a tube voltage of 120 kVp, and a tube current of 400 mA. All patients were scanned in the prone position, except for elderly or physically debilitated patients who were unable to maintain this position. In our institution, all rectal cancer contours, including the final treatment contours in the first component and GT contours in the second component, were delineated according to the Valentini and RTOG guidelines.^4^, ^23^ To ensure quality, they underwent a strict review process: initial contouring by junior/mid‐level oncologists, secondary modification by senior oncologists, and final confirmation during departmental rounds.

#### 2.2.1 Quantitative geometric comparison and failure rate statistics

The delineated structures of 150 rectal cancer cases included the CTV, bladder, femur, intestine, colon, and perineum. Table 1 specifically depicts the deployment status of the automatic contouring system of three cohorts and phases, the corresponding automatic contours and final treatment contours, as well as their abbreviations.

**TABLE 1:** Summarized contour‐related characteristics of 150 patients in three phases.

Cohort	Deployment status and phase	Auto contour	Final treatment contour
Pre‐implementation cohort (n1 = 50)	Not yet implemented (before May 2018)	C_Auto1, n1_ (retrospectively generated)	C_MAN, n1_ (directly collected)
Post‐implementation cohort (n2 = 50)	Implement the first‐generation automatic contouring system (May 2018 to November 2024)	C_Auto1, n2_ (directly collected)	C_Auto1, rev, n2_ (directly collected)
Post‐update cohort (n3 = 50)	Implement the second‐generation automatic contouring system (after December 2024)	C_Auto2, n3_ (directly collected)	C_Auto2, rev, n3_ (directly collected)

Abbreviation: CAuto1, n1 and CMAN, n1 represent unedited‐automatic contours generated by the first‐generation system and fully manual contours in the pre‐implementation cohort, respectively. CAuto1, n2 and CAuto1, rev, n2 denote unedited‐automatic contours generated by the first‐generation system and manually reviewed contours based on CAuto1 in the post‐implementation cohort, respectively. CAuto2, n3 and CAuto2, rev, n3 represent unedited‐automatic contours generated by the second‐generation system and manually reviewed contours based on CAuto2 in the post‐update cohort, respectively. The contours used for clinical delineation can be directly collected, while those not used for clinical delineation at that time were retrospectively generated.

In routine clinical practice, the workflow for final treatment contours shows some variation according to the implementation phase. In the pre‐implementation phase, the contours were initially delineated manually by oncologists with 5–10 years of clinical experience. In the post‐implementation and post‐update phases, oncologists modified the auto‐generated contours based on the initial automatic segmentation. Regardless of the initial contouring method, all contours across the three phases were systematically reviewed and modified by senior oncologists with more than 15 years of experience, and ultimately authorized during departmental clinical rounds prior to treatment delivery. Consequently, comparing auto‐generated contours to these final treatment contours provides a direct measurement of the real‐world clinical editing burden.

To evaluate the impact of implementing the first‐generation automatic contouring system on contour generation, we compared the geometric similarities between the Auto1‐generated contours and the final treatment contours in the pre‐ and post‐implementation cohorts. In the pre‐implementation cohort, we calculated the geometric similarity between the retrospective Auto1‐generated contours (C_Auto1, n1_) and the finally manual contours (C_MAN, n1_). This similarity reflects the agreement between Auto1‐generated output and human delineation. In the post‐implementation cohort, the final treatment contours (C_Auto1, rev, n2_) were formed after oncologists reviewed and modified the Auto1‐generated contours (C_Auto1, n2_). This similarity reflects the concordance between the clinically approved final contours after editing and the initial Auto1‐generated contours. By comparing the difference in similarity metrics between these two cohorts, we were able to quantify the impact of the automatic contouring system's introduction on oncologist's contouring habits and burden. It reveals the shift in clinical acceptance of and reliance on AI contours within the actual workflow. Furthermore, to investigate the impact of the system update, the similarities between the initial AI‐generated contours and the final edited treatment contours were evaluated in post‐implementation phase and post‐update phase. Subsequently, these metrics from the first‐generation system (C_Auto1, n2_ vs. C_Auto1, rev, n2_) were compared with those from the second‐generation system (C_Auto2, n3_ vs. C_Auto2, rev, n3_). Appendix A presents the geometric similarity metrics of Dice similarity coefficient (DSC)^24^ and mean distance to agreement (MDA)^25^, ^26^ and the statistical methods.

To separately identify failure rates of the two generations of automatic contouring systems, we set a three‐sigma limit of the DSC value to determine the failure rates, with the presumption that 99.7% of results should fall within this range.^27^ The oncologist and the physicist jointly analyzed the reasons for failure.

#### 2.2.2 Analysis of inter‐observer consistency, efficiency, and accuracy

To evaluate the impact of two generations of automatic contouring systems on oncologist workload, contouring efficiency, accuracy, and inter‐observer consistency, this retrospective study randomly included 21 patients with rectal cancer who had undergone preoperative radiotherapy. Six radiation oncologists, each with over five years of experience in rectal cancer radiotherapy contouring, participated in the study.

To minimize bias, the six oncologists were divided into three groups of two, ensuring comparable clinical experience across groups. The 21 cases were randomly divided into three subsets of seven. By drawing lots, the assignment of the three contouring methods (fully manual, first‐generation auto‐assisted, and second‐generation auto‐assisted) to the oncologist groups was randomly determined for each subset. Crucially, the three methods for any given case were performed by different oncologist groups. Because no oncologist contoured the same case under multiple methods, recall bias was eliminated, negating the need for a washout period. Furthermore, all images were strictly anonymized to prevent any potential recognition bias, and the presentation order of cases was randomized. Although blinding to the contouring method was inherently unfeasible, oncologists were completely blinded to the ground truth and the contours generated by their peers. All contours were refined to a clinically acceptable standard.

To assess efficiency, we recorded the contouring time separately for the CTV and OARs. Regarding inter‐observer consistency, we calculated the DSC value between contours from the two oncologists within the same contouring method. To assess contouring accuracy, we computed the DSC value between the contours from each method and the final treatment contours in our institution, which were generated through the same multistep clinical review process described above, serving as the GT.

Additionally, to evaluate the clinical acceptability of the contours, a 5‐point Likert scale^28^, ^29^ (5: excellent/no edits needed; 4: good/minor edits needed; 3: acceptable/moderate edits needed; 2: poor/major edits needed; 1: unusable) was employed. This clinical evaluation consisted of two primary parts. The first part aimed to validate whether the final contours submitted by the six participating oncologists, regardless of whether manually delineated or modified from the two generations of automatic contours, met clinically acceptable standards. The second part was designed to directly evaluate and compare the unedited contours generated by the two generations of automatic contouring systems to determine which system demonstrated superior clinical feasibility. To ensure objectivity, two senior radiation oncologists independently evaluated the contours in a completely blinded manner (blinded to both the contouring method and contour origin). The final clinical acceptability score for each contour was calculated as the mean of the scores provided by the two senior oncologists.

### 2.3 Statistical analysis

For baseline characteristics among the three cohorts, global tests were performed for each variable. The Kruskal‐Wallis test was used for the continuous variable. For categorical variables, the chi‐square test was applied when all expected cell frequencies were ≥ 5, otherwise, the Fisher's exact test was used. A two‐sided *P*‐value > 0.05 was considered to indicate no statistically significant difference among the cohorts, suggesting balanced baseline characteristics.

For geometric similarity comparisons (DSC and MDA) between independent cohorts (pre‐ vs. post‐implementation, and post‐implementation vs. post‐update), the Mann‐Whitney U test was conducted.

For comparisons among the three contouring methods (manual, Auto1‐assisted, and Auto2‐assisted) using the 21‐patient dataset, the Kruskal‐Wallis test was first performed to assess overall differences. When a statistically significant difference was detected, pairwise comparisons between the contouring methods were subsequently performed using the Wilcoxon signed‐rank test.

The Mann‐Whitney U test and Wilcoxon signed‐rank test were used for independent samples and paired samples, respectively, and neither test requires normal distribution assumptions. For all tests, a *P*‐value < 0.05 was considered statistically significant.

## 3 RESULTS

The three cohorts across the three clinical phases were well balanced in baseline characteristics, including age, sex, clinical stage, and tumor distance from the anal verge (all *P* > 0.05, detailed in Appendix B).

**FIGURE 2:**
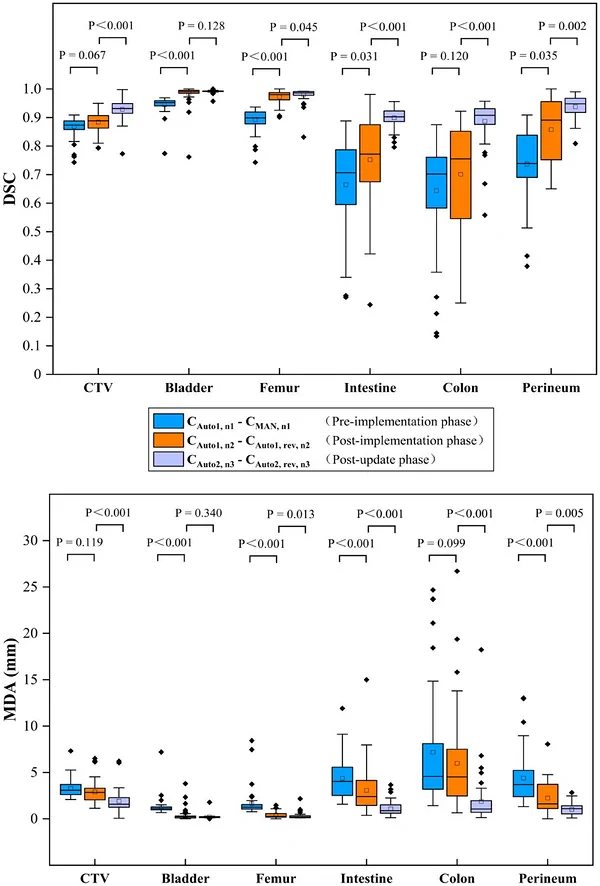
Longitudinal comparison of geometric similarity metrics (DSC and MDA) for CTV and OARs across three clinical phases: pre‐implementation, post‐implementation, and post‐update.

Figure 2 presents the boxplots of DSC and MDA comparisons across the three cohorts for CTV and OARs. Appendix C and Appendix D list the specific values. Across the three phases, the similarity metrics demonstrated a continuous progressive improvement, characterized by progressively higher DSC values and lower MDA values from pre‐implementation to post‐implementation and subsequently to post‐update.

Regarding before and after implementation of the automatic contouring system, most of the structures showed significantly higher DSC values and lower MDA values in the post‐implementation cohort compared with those in the pre‐implementation cohort. The mean DSC values before and after implementing were 0.87 ± 0.04 and 0.88 ± 0.04 for CTV (*P* = 0.067) and 0.80 ± 0.06 and 0.88 ± 0.05 for OARs (*P* < 0.001), respectively. The geometric similarity between the unedited‐automatic contours and the AI‐assisted final contours in the post‐implementation cohort was higher than that between the unedited‐automatic contours and the completely manual final contours in the pre‐implementation cohort. Therefore, after implementing the automatic contouring system, the oncologists may have received guidance from the system when delineating CTV and OARs.

Regarding before and after update of the automatic contouring system, all structures demonstrated higher DSC values and lower MDA values in the post‐update cohort than those in the post‐implementation cohort, with the vast majority of these improvements being statistically significant. The mean DSC values before and after updating were 0.88 ± 0.04 and 0.93 ± 0.04 for CTV (*P* < 0.001) and 0.88 ± 0.05 and 0.95 ± 0.02 for OARs (*P* < 0.001), respectively. Therefore, after iterative updating, the similarities between the automatic results and the AI‐assisted final contours in the post‐update cohort were superior to those in the post‐implementation cohort.

Appendix E presents the control charts of the automatic contouring systems’ failure rates for different structures during the three phases. The failure rates of the first‐generation system for CTV, bladder, femur, intestine, colon, and perineum were 1.67%, 3.67%, 3.33%, 4.33%, 3.33%, and 3.47%, whereas those of the second‐generation system were 0.67%, 0.67%, 1.67%, 0.33%, 0.00%, and 0.50%, respectively. After automatic contouring system update, the mean failure rate decreased from 3.30% to 0.64%. Overall, the increased similarity and reduced failure rate indicate that the second‐generation automatic contouring system lowered the extent of oncologist editing and delivered superior performance.

For the first‐generation and second‐generation DL‐based automatic contouring methods (Auto1‐ and Auto2‐assisted methods, respectively), the total time was divided into two parts. The first was the system invocation time, which included the time for invoking the system, transmitting CT images to the TPS, generating the auto‐contours, and returning these contours to the TPS. The second was the time for reviewing and revising these auto‐contours by radiation oncologists. Table 2 summarizes the time required for each step of the three methods. The total time significantly differed between the three contouring methods (*P* < 0.05), with the Auto2‐assisted method being the fastest (16.97 ± 5.52 min) and manual contouring being the most time‐consuming (41.15 ± 10.43 min). Pairwise comparisons of inter‐observer consistency, contouring accuracy, and efficiency among the three contouring methods were performed only after the global test showed statistical significance. The results of Kruskal‐Wallis global test are provided in the Appendix F.

**TABLE 2:** Summary of the time of all required steps for each method.

		Time (min) of the three methods			*P*‐value		
Step	Structure	Auto1‐assisted	Auto2‐assisted	MAN	Auto1 vs Auto2	Auto1 vs MAN	Auto2 vs MAN
automatic contouring system invocation	N/A	2.50 ± 0.86	1.89 ± 0.45	N/A	< 0.001	N/A	N/A
manual revising or fully manual contouring	CTV	11.28 ± 4.60	8.32 ± 3.29	22.90 ± 8.39	< 0.001	< 0.001	< 0.001
	OARs	7.96 ± 4.63	6.76 ± 4.37	18.25 ± 8.56	0.01	< 0.001	< 0.001
Total	N/A	21.74 ± 5.19	16.97 ± 5.52	41.15 ± 10.43	< 0.001	< 0.001	< 0.001

Table 3 presents the comparisons of inter‐observer consistency and contouring accuracy among the three methods, as well as the statistical differences between each pair of methods. The Auto2‐assisted contouring group demonstrated significantly higher inter‐observer consistency than the other groups (all: *P* < 0.05). The mean DSC values in the Auto2‐assisted, Auto1‐assisted, and manual contouring groups were 0.95 ± 0.03, 0.93 ± 0.02, and 0.92 ± 0.04 for CTV, and 0.92 ± 0.16, 0.85 ± 0.20, and 0.83 ± 0.20 for OARs, respectively. Regarding contouring accuracy, structures assisted by the second‐generation contouring system also showed better agreement with GT than those by the first‐generation system and manual contours, with most comparative *P*‐values < 0.001. The mean DSC values in the Auto2‐assisted, Auto1‐assisted, and manual contouring groups were 0.94 ± 0.03, 0.93 ± 0.02, and 0.91 ± 0.03 for CTV, and 0.92 ± 0.14, 0.90 ± 0.14, and 0.85 ± 0.17 for OARs, respectively.

**TABLE 3:** Comparisons of inter‐observer consistency and oncologists’ contouring accuracy among the three contouring methods.

		CTV	Bladder	Femur L	Femur R	Intestine	Colon
Inter‐observer consistency comparison	DSC_Group _Auto1_	0.93 ± 0.02	0.98 ± 0.02	0.90 ± 0.19	0.90 ± 0.19	0.76 ± 0.16	0.70 ± 0.22
	DSC_Group _Auto2_	0.95 ± 0.03	1.00 ± 0.01	0.94 ± 0.20	0.94 ± 0.20	0.89 ± 0.11	0.83 ± 0.19
	DSC_Group _MAN_	0.92 ± 0.04	0.96 ± 0.02	0.92 ± 0.04	0.92 ± 0.05	0.72 ± 0.22	0.64 ± 0.25
	*P*‐value (Group _Auto1_ *vs* Group _Auto2_)	0.024	0.003	0.001	<0.001	0.012	0.010
	*P*‐value (Group _Auto1_ *vs* Group _MAN_)	0.114	0.001	0.045	0.095	0.913	0.108
	*P*‐value (Group _Auto2_ *vs* Group _MAN_)	0.022	<0.001	0.017	0.039	0.007	0.006
Contouring accuracy comparison	DSC_Auto1	0.93 ± 0.02	0.98 ± 0.01	0.94 ± 0.05	0.95 ± 0.03	0.83 ± 0.16	0.77 ± 0.18
	DSC_Auto2	0.94 ± 0.03	0.99 ± 0.01	0.95 ± 0.14	0.97 ± 0.02	0.86 ± 0.14	0.81 ± 0.19
	DSC_MAN	0.91 ± 0.03	0.97 ± 0.02	0.93 ± 0.04	0.93 ± 0.04	0.70 ± 0.21	0.66 ± 0.17
	*P*‐value (Auto1 *vs* Auto2	0.288	<0.001	0.004	0.002	0.425	0.266
	*P*‐value (Auto1 *vs* MAN)	<0.001	<0.001	0.049	0.001	0.008	0.007
	*P*‐value (Auto2 *vs* MAN)	<0.001	<0.001	<0.001	<0.001	<0.001	0.001

Abbreviation: Same as the description in Figure 1.

Clinical evaluation was performed in two parts, with detailed scoring data provided in Appendix G. First, among the 126 final contours produced by the six participating oncologists, 99.2% (125/126) received a Likert score of ≥ 4, with only one contour rated 3.5, indicating that nearly all final contours produced by the six oncologists required only minor or no further edits. Second, the unedited contours generated by the two auto‐contouring systems were directly compared. Auto2 demonstrated significantly better clinical acceptability than Auto1, with a higher mean Likert score (4.02 ± 0.25 vs. 3.26 ± 0.49, *P* < 0.001). In addition, 95.2% (20/21) of Auto2‐generated contours achieved a score of ≥ 4, compared with 28.6% (6/21) for Auto1. Auto2 scored higher than Auto1 in 15 cases, while the remaining 6 cases showed identical scores.

## 4 DISCUSSION

The core contribution of this study is the longitudinal, systematic evaluation of how a DL‐based automatic contouring system, as well as its iterative updates, performs in real‐world rectal cancer radiotherapy. This longitudinal comparison enabled us to evaluate the geometric performance of two different AI models while also examining their impacts on clinical workflows, contour quality, and failure modes across different phases.

A series of studies developed automatic contouring models in rectal cancer radiotherapy. For instance, Men et al.^11^ developed a Deep Dilated Convolutional Neural Network–based model for the auto‐segmentations of CTV with a mean DSC value of 0.877 in patients with rectal cancer. Rasmus et al.^30^ designed a 3D V‐net architecture derived from the U‐Net, with a DSC reaching 0.90. Yin et al.^31^ improved the conventional U‐Net and proposed Flex U‐Net, which used a register model to correct the manual annotation–induced noise; their framework finally achieved DSC values of 0.817 ± 0.071, 0.930 ± 0.076, 0.927 ± 0.03, and 0.925 ± 0.03 for CTV, bladder, femur head‐L, and femur head‐R, respectively. Song et al.^32^ reported a DSC value of 0.88 for CTV in rectal cancer; both the automatic contours generated by the first‐generation system (DSC: 0.88) and the second‐generation system (DSC: 0.93) demonstrated superior performance compared with those in other studies.

Moreover, Lee et al.^33^ evaluated the impact of a DL‐based auto‐contouring system for breast radiotherapy. They repredicted contours for the pre‐implementation group, collected auto‐contours in the post‐implementation group, and compared them with the final contours, which were generated by editing the auto‐contours. Results showed that the similarity metrics between auto‐generated and final edited contours significantly increased, indicating growing clinical reliance on the automatic system. We employed a similar methodology, but aside from assessing the impact of implementing an automatic contouring system, we also evaluated the effect of the system's iterative update. Geometric metrics were compared longitudinally between the pre‐ and post‐implementation cohorts to evaluate the clinical impact of the auto‐contouring system. The DSC values between C_Auto1, n1_ and C_MAN, n1_ in the pre‐implementation cohort were lower than those between C_Auto1, n2_ and C_Auto1, rev, n2_ in the post‐implementation cohort for all structures, with statistically significant differences in all structures except the CTV and colon. The final contours obtained after reviewing and revising the first‐generation automatic contours were closer to the auto‐contours than the manually delineated contours. This finding suggests that with the introduction of the auto‐contouring system, oncologists’ contouring and editing were more guided by the automatic system. Furthermore, the clinical impact of the iterative update of the automatic contouring system was evaluated through a longitudinal geometric comparison between the post‐implementation and post‐update cohorts. The auto‐contours generated by the second‐generation system showed significantly better agreement with the final contours compared with those produced by the first‐generation system, with all structures, except the bladder, showing statistically significant differences. Therefore, contouring accuracy substantially improved after the system update. The three cohorts were generally comparable in baseline characteristics, with no significant differences in age, sex, clinical stage, or tumor distance from the anal verge, suggesting that the observed performance differences were unlikely to be explained by patient composition.

Each DL‐based automatic contouring algorithm may encounter failure modes. Comprehensive clinical quality assurance is achievable only when the possible failure modes of the automatic system are well understood. While analyses of automatic contouring failure modes have been reported for breast cancer^33^ and head and neck cancer,^27^ no similar research exists for rectal cancer. In the present study, we analyzed the failure modes of two generations of automatic contouring systems. Failure modes and causes identified in this study provide valuable feedback for future model upgrades.

The following failure modes for CTV contouring were identified. (i) No integration of multi‐modal imaging data: First, the AI model trained solely on CT images misidentified the levator ani muscle as lymph node involvement, a distinction that could be clarified using MRI simulation images. Second, the AI model could not extend the GTV in a nodal disease to cover the para‐aortic lymphatic drainage area, a step that radiation oncologists routinely take when diagnostic imaging suggests metastasis. Therefore, incorporating the patients’ medical history and all available imaging reports, including those obtained by CT and MRI, is essential. (ii) Misidentification of adjacent anatomical structures: The AI model incorrectly incorporated the bowels adjacent to the GTV into the tumor target area, thereby overestimating the CTV. (iii) Deviation in patient positioning: Preoperative patients with rectal cancer are typically scanned in the prone position. However, older or physically weak patients who cannot maintain this position may be scanned supine, thereby introducing variability that affected CTV prediction. The following failure modes were identified for bladder contouring. (i) Misidentification of adjacent anatomical structures: AI mistakenly interpreted the hyperplastic prostate adjacent to the bladder as the bladder. (ii) Deviation in patient positioning: This failure mode is similar to that of the CTV mentioned above. (iii) Specificity of patient anatomy: Poor bladder filling of patients led to deviations in bladder contouring prediction. Currently, a failure mode has been discovered for the femur, that is, the misidentification of adjacent anatomical structures. The AI model misidentified the pelvic bone or femoral neck adjacent to the femoral head as the femoral head. As for the intestine and colon, the main failure mode was the misidentification of adjacent anatomical structures, and it was divided into three scenarios. First, the AI model mistakenly confused the small intestine and the colon. Second, the AI model mistook the muscles on both colonic sides for the colon. Third, AI mode vaguely recognized the rectum‐colon boundary. Either the first‐generation model alone or both generations manifested the abovementioned failure modes. Nevertheless, the failure rate in the second‐generation model decreased by approximately 80.6% compared with that in its predecessor. Subsequent studies should focus on refining the model by addressing these recurrent failure modes to further enhance auto‐contouring performance.

Furthermore, contouring evaluations from six radiation oncologists were incorporated to validate the impact of implementing and updating the automatic contouring system. This multi‐observer study robustly demonstrated that the second‐generation system significantly enhanced clinical workflow compared with both the first‐generation system and manual contouring. Aside from being able to reduce the total contouring time by approximately 58.8% compared with fully manual contouring, this Auto2‐assisted method achieved the shortest manual editing time, highlighting its efficiency. More importantly, it simultaneously improved both inter‐observer consistency and contouring accuracy, showing statistical significance in most comparisons. Notably, the blinded clinical evaluation provided further support for the real‐world usability of the contours: the final contours refined by the six oncologists were almost all clinically acceptable, and the second‐generation system achieved higher acceptability scores than the first‐generation system. Taken together, the system update provides superior clinical utility, offering substantial gains in efficiency, reliability, and clinical feasibility.

Two major limitations of this study should be acknowledged. First, although the present study comprehensively evaluated contouring efficiency, geometric performance, inter‐observer consistency, and blinded clinical acceptability, dosimetric evaluation was not included in the current work. Previous studies^34^, ^35^ have shown that contour discrepancies may result in measurable differences in target coverage and OARs dose estimation, indicating that geometric similarity does not necessarily ensure dosimetric equivalence. In the present study, the favorable findings across multiple clinically relevant dimensions support the value of system implementation and iterative updating. Future work is still needed to perform patient‐specific dosimetric analyses that directly quantify the impact of contour differences. Second, the current study compared different patient cohorts across three phases. This longitudinal design was necessary to accurately reflect the real‐world evolution of clinical workflows. However, this approach may introduce confounding effects due to individual patient differences across the distinct cohorts. To address this issue, we statistically evaluated the baseline characteristics, confirming that potential confounding factors were well‐balanced among the cohorts. Potential time‐related biases were minimized because CT imaging protocols, patient positioning, reference guidelines, and reference guidelines remained uniform throughout the study period. Furthermore, a substudy in which six oncologists re‐delineated 21 cases using all three methods on the same patient set demonstrated consistent improvements in inter‐observer consistency, contouring accuracy, and time efficiency, providing supportive evidence for our main findings.

## 5 CONCLUSION

This longitudinal real‐world study highlights the transformative value of implementing and updating a DL‐based automatic contouring system in rectal cancer radiotherapy. While the initial implementation provided essential guidance to oncologists during contouring, the subsequent system update significantly reduced their workload, minimized inter‐observer variation, and improved overall contouring efficiency.

## AUTHOR CONTRIBUTIONS

Wang NY, Xu TZ, Dai JR, Tang Y and Men K have made substantial contributions to the conception or design of the work. Wang NY, Kang YJ, Liang YT, Wang WY and Nie WS have made substantial contributions to the acquisition, analysis. Wang NY, Xu TZ and Men K have made substantial contributions to the interpretation of data. Wang NY, Kang YJ and Men K have made substantial contributions to the creation of new software used in the work. Wang NY, Dai JR, Tang Y and Men K have drafted the work or substantively revised it. All authors read and approved the final manuscript.

## CONFLICT OF INTEREST STATEMENT

The authors declare no conflicts of interest.

## ETHICS STATEMENT

The retrospective study was in accordance with the Declaration of Helsinki, and was approved by the review board of the National Cancer Center/Cancer Hospital, Chinese Academy of Medical Sciences. Consent to participate was obtained from all of the participants in this study. Human Ethics and Consent to Participate declarations: not applicable.

## Supporting information




Supporting Information

## Data Availability

All supporting data are included as supplementary files with this manuscript. Additional raw data can be obtained from the corresponding author upon reasonable request.
