# Supplementary material for: Real‐world clinical impact of implementing and updating a deep learning‐based automatic contouring system in rectal cancer radiotherapy
Source: J Appl Clin Med Phys. 2026 Aug 28;27(9):e70768. doi: 10.1002/acm2.70768 (PMC13525157; doi:10.1002/acm2.70768)
Supplement: Supplementary file 1 [file ACM2-27-e70768-s001.docx]

**Appendices**

**Appendix A: Introduction of the geometric similarity metrics of DSC and MDA**

Geometric accuracy was quantified using the MIM software. The formulas are as follows:

$\text{DSC =}\frac{\text{2|}\text{A}\text{ ∩ }\text{B}\text{|}}{\text{|}\text{A}\text{| + |}\text{B}\text{|}}$	Eq. (A.1)

where A and B denote sets of voxels that define volumes within two contours. DSC provides a unitless measure of the volumetric overlap of two contours, where DSC = 0 indicates no overlap and DSC = 1 indicates perfect overlap.

$\text{MDA(A, B) =}\frac{\text{h(}\text{A}\text{,}\text{B}\text{) + h(}\text{B}\text{,}\text{A}\text{)}}{\text{2}}$	Eq. (A.2)

$\text{h(A,B) =}\underset{\text{a}\text{∈}\text{A}}{\text{mean}}\text{\{}\min_{\text{b}\text{∈}\text{B}}\text{\{ }\text{d}\text{ (}\text{a}\text{,}\text{b}\text{) \} \}}$	Eq. (A.3)

The mean 3D distances between contours A and B were quantified using MDA, which was introduced to alleviate sensitivity to outliers. Additionally, *d* (a, b) represents the 3D Hausdorff distance between point *a* from contour A and point *b* from contour B. For a perfect overlap, the MDA value is 0; for an imperfect overlap, it is increased.

Statistical data were analyzed using Wilcoxon signed-rank test to compare DSC and MDA values measured in auto-contours and final treatment contours. *P*-values below 0.05 were considered statistically significant.

**Appendix B: Baseline characteristics of the three cohorts of 150 patients**

Table B. Baseline characteristics of patients across the three clinical phases.

| Characteristics | Pre-implementation (n1=50) | Post-implementation (n=50) | Post-update  (n=50) | P value |
| --- | --- | --- | --- | --- |
| Age, years |  |  |  | 0.690 |
| Median (range) | 56 (31-89) | 58 (39-81) | 56 (43-73) |  |
| Sex, n (%) |  |  |  | 0.131 |
| Male | 31 (62) | 32 (62) | 22 (44) |  |
| Female | 19 (38) | 19 (38) | 28 (56) |  |
| Clinical stage, n (%) |  |  |  | 0.812 |
| II | 4 (8) | 4 (8) | 2 (4) |  |
| III | 42 (84) | 43 (86) | 44 (88) |  |
| IV | 4 (8) | 3 (6) | 4 (8) |  |
| Tumor distance from anal verge, n (%) |  |  |  | 0.060 |
| ≤5 cm | 8 (16) | 14 (28) | 11 (22) |  |
| >5-10 cm | 24 (48) | 23 (46) | 33 (66) |  |
| ≥10 cm | 18 (36) | 13 (26) | 6 (12) |  |

**Appendix C: DSC and MDA comparisons before and after implementation of the automatic contouring system**

Table C. Comparison of similarity metrics before and after implementing the automatic contouring system for CTV and OAR structures. DSC and MDA measurements are summarized using mean ± SD.

| Structure | DSC | | | MDA (mm) | | |
| --- | --- | --- | --- | --- | --- | --- |
|  | C_Auto1, n1_ - C_MAN, n1_  (pre-implementation cohort) | C_Auto1, n2_ - C_Auto1, rev, n2_  (post-implementation cohort) | P value | C_Auto1, n1_ - C_MAN, n1_  (pre-implementation cohort) | C_Auto1, n2_ - C_Auto1, rev, n2_  (post-implementation cohort) | P value |
| CTV | 0.87 ± 0.04 | 0.88 ± 0.04 | 0.067 | 3.26 ± 0.90 | 2.90 ± 1.17 | 0.119 |
| Bladder | 0.95 ± 0.03 | 0.98 ± 0.04 | ＜0.001 | 1.27 ± 0.91 | 0.36 ± 0.62 | ＜0.001 |
| Femur | 0.89 ± 0.04 | 0.97 ± 0.02 | ＜0.001 | 1.64 ± 1.40 | 0.40 ± 0.33 | ＜0.001 |
| Intestine | 0.66 ± 0.16 | 0.75 ± 0.15 | 0.031 | 4.36 ± 2.28 | 3.05 ± 2.48 | ＜0.001 |
| Colon | 0.64 ± 0.18 | 0.70 ± 0.18 | 0.120 | 7.17 ± 5.98 | 5.98 ± 5.08 | 0.099 |
| Perineum | 0.74 ± 0.13 | 0.86 ± 0.11 | 0.035 | 4.39 ± 3.04 | 2.24 ± 1.80 | ＜0.001 |

**Appendix D: DSC and MDA comparisons before and after updating the automatic contouring system**

Table D. Comparison of similarity metrics before and after updating the automatic contouring system for CTV and OAR structures. DSC and MDA measurements are summarized using mean ± SD.

| Structure | DSC | | | MDA (mm) | | |
| --- | --- | --- | --- | --- | --- | --- |
|  | C_Auto1, n2_ - C_Auto1, rev, n2_  (post-implementation cohort) | C_Auto2, n3_ - C_Auto2, rev, n3_  (post-update cohort) | P value | C_Auto1, n2_ - C_Auto1, rev, n2_  (post-implementation cohort) | C_Auto2, n3_ - C_Auto2, rev, n3_  (post-update cohort) | P value |
| CTV | 0.88 ± 0.04 | 0.93 ± 0.04 | ＜0.001 | 2.90 ± 1.17 | 1.90 ±1.12 | ＜0.001 |
| Bladder | 0.98 ± 0.04 | 0.99 ± 0.01 | 0.128 | 0.36 ± 0.62 | 0.21 ± 0.23 | 0.340 |
| Femur | 0.97 ± 0.02 | 0.98 ± 0.02 | 0.045 | 0.40 ± 0.33 | 0.29 ± 0.31 | 0.013 |
| Intestine | 0.75 ± 0.15 | 0.90 ± 0.03 | ＜0.001 | 3.05 ± 2.48 | 1.07 ± 0.79 | ＜0.001 |
| Colon | 0.70 ± 0.18 | 0.89 ± 0.07 | ＜0.001 | 5.98 ± 5.08 | 1.87 ± 2.73 | ＜0.001 |
| Perineum | 0.86 ± 0.11 | 0.94 ± 0.04 | 0.002 | 2.24 ± 1.80 | 1.02 ± 0.63 | 0.005 |

**Appendix E: Control charts and failure rates statistics**

Figure E.1 shows the three-sigma limit results of the DSC values for the first- and second-generation automatic contouring algorithms in the pre-implementation cohort (n1 = 50). Similarly, Figure E.2 and E.3 show the control charts in the post-implementation cohort (n2 = 50) and the post-update cohort (n3 = 50), respectively. The green line shows the mean of the points, while the red lines denote the upper and lower control limits (UCL and LCL, respectively). In these figures, for each structure, the upper part corresponds to the first generation of automatic contouring system, and the lower part corresponds to the second generation of automatic contouring system. The failure rates of both systems are evaluated using the same criteria.

Fig. E.1 Three-sigma limit results of the DSC values for two generations of automatic contouring systems in the pre-implementation cohort (n1 = 50).

V

Fig. E.2 Three-sigma limit results of the DSC values for two generations of automatic contouring systems in the post-implementation cohort (n2 = 50).

Fig. E.3 Three-sigma limit results of the DSC values for two generations of automatic contouring systems in the post-update cohort (n3 = 50).

**Appendix F: Global statistical comparisons among the three contouring methods**

Table F. Global Kruskal-Wallis test results for efficiency, inter-observer consistency, and contouring accuracy among the three contouring methods.

| Characteristics | Structures | H value | P value |
| --- | --- | --- | --- |
| Total contouring time | N/A | 77.67 | ＜0.001 |
| Inter-observer consistency | CTV | 10.29 | 0.006 |
|  | Bladder | 26.70 | ＜0.001 |
|  | Femur L | 13.76 | ＜0.001 |
|  | Femur R | 14.39 | ＜0.001 |
|  | Intestine | 9.66 | 0.008 |
|  | Colon | 9.61 | 0.008 |
| Contouring accuracy comparison | CTV | 11.04 | ＜0.001 |
|  | Bladder | 28.07 | ＜0.001 |
|  | Femur L | 23.52 | ＜0.001 |
|  | Femur R | 26.45 | ＜0.001 |
|  | Intestine | 18.13 | ＜0.001 |
|  | Colon | 18.22 | ＜0.001 |

**Appendix G: Detailed Likert scoring data for each contour**

Table G. Likert scores for each contour. Scores for the six oncologists' final contours (columns A - F) and for the unedited Auto1- and Auto2-generated contours were each averaged from two independent senior oncologists.

| Patient | Oncologist A’s contours | Oncologist B’s contours | Oncologist C’s contours | Oncologist D’s contours | Oncologist E’s contours | Oncologist F’s contours | Auto1-generated  contours | Auto2-generated  contours |
| --- | --- | --- | --- | --- | --- | --- | --- | --- |
| 1 | 5 | 5 | 4 | 5 | 5 | 5 | 3 | 4 |
| 2 | 4 | 5 | 4 | 5 | 5 | 4 | 3 | 4 |
| 3 | 5 | 5 | 4 | 4 | 5 | 5 | 3 | 4 |
| 4 | 5 | 5 | 4 | 5 | 5 | 5 | 3 | 5 |
| 5 | 5 | 5 | 5 | 5 | 5 | 5 | 2.5 | 3.5 |
| 6 | 4 | 5 | 4 | 5 | 5 | 4 | 4 | 4 |
| 7 | 4 | 4 | 4 | 5 | 5 | 5 | 3 | 4 |
| 8 | 5 | 5 | 4 | 5 | 5 | 4 | 3 | 4 |
| 9 | 4 | 4 | 4 | 4 | 4 | 4 | 4 | 4 |
| 10 | 4.5 | 4 | 4 | 4 | 4 | 4 | 4 | 4 |
| 11 | 3.5 | 4 | 4 | 5 | 4 | 4 | 3 | 4 |
| 12 | 4 | 4 | 4 | 5 | 5 | 5 | 3 | 4 |
| 13 | 4 | 5 | 5 | 5 | 5 | 5 | 4 | 4 |
| 14 | 4 | 5 | 5 | 5 | 5 | 5 | 3 | 4 |
| 15 | 5 | 5 | 4 | 5 | 5 | 4 | 3 | 4 |
| 16 | 4 | 5 | 4 | 5 | 5 | 4 | 3 | 4 |
| 17 | 4 | 4 | 4 | 5 | 4 | 4 | 3 | 4 |
| 18 | 5 | 4 | 5 | 5 | 4 | 4 | 4 | 4 |
| 19 | 5 | 5 | 5 | 5 | 4 | 4 | 4 | 4 |
| 20 | 5 | 4 | 4 | 4 | 4 | 4 | 3 | 4 |
| 21 | 4 | 4 | 4 | 4 | 5 | 4 | 3 | 4 |
